# Screening of fabry disease in patients with left ventricular hypertrophy: a single-center study in Mexico

**DOI:** 10.1186/s13023-026-04506-z

**Published:** 2026-07-25

**Authors:** Dante Palacios Gutierrez, Gabriela Meléndez-Ramirez, Edith Liliana Posada Martínez, Blanca Rebeca Ibarra-Ibarra, Solange Gabriela Koretzky, Maria Cecilia Escalante Seyffert, Francisco Javier Roldán Gómez, Aloha Meave Gonzalez Ramirez, Lucía de Montserrat Sabido González, Daniel Vera Ibarra, Juan Luis Ortiz Herrera, Karla Vázquez Fernández, Daniela Ramirez de Jesús, Enrique Alexander Berrios Barcenas

**Affiliations:** 1 Cardiomyopathy Clinic, Instituto Nacional de Cardiología Ignacio Chávez, Juan Badiano 1, Belisario Dominguez Sección XVI, Mexico City, 14080 Mexico; 2 Cardiac Magnetic Resonance Department, Instituto Nacional de Cardiología Ignacio Chávez, Mexico City, Mexico; 3 Echocardiography Department, Instituto Nacional de Cardiología Ignacio Chávez, Mexico City, Mexico; 4 Cardiogenetics Clinic, Instituto Nacional de Cardiología Ignacio Chávez, Mexico City, Mexico; 5 Publications Department, Instituto Nacional de Cardiología ignacio Chávez, Mexico City, Mexico; 6 Research Department, Hospital Infantil de México Federico Gómez, Mexico City, Mexico; 7 Horizon Clinical Research Group, Pulmonology Division, HCA North Cypress, Houston, TX USA

**Keywords:** Fabry disease, Left ventricular hypertrophy, Cardiovascular diseases

## Abstract

**Background:**

Fabry disease (FD) is an X-linked sphingolipidosis caused by enzyme α-Galactosidase A (α-Gal A) deficiency due to pathogenic variants in *GLA* gene. Progressive glycosphingolipid accumulation leads to subsequent myocyte injury, which frequently induces concentric remodeling and manifests as left ventricular hypertrophy (LVH). This study aims to describe the prevalence of FD in patients with LVH diagnosis in a Mexican population.

**Methods:**

Consecutive and unrelated patients with echocardiographic evidence of left ventricular hypertrophy (LVH) without known cause were screened for FD in a Mexican cardiology referral center. Globotriaosylsphingosine (lyso-Gb3) levels and α-Gal A activity were measured using the dry blood spot method in all male individuals. Patients with positive dried blood spot testing and female patients were confirmed by single-gene sequencing.

**Results:**

205 patients with LVH (48.5 ± 18.7 years) were included. Of these, 53% were male, 60% were in NYHA functional class I, and 43% were asymptomatic. Dyspnea was the most frequently reported symptom, occurring in 26% of cases. Among study participants, three women were diagnosed with FD (1.5%). *GLA* gene sequencing revealed two pathogenic variants, NM_000169.3(*GLA*):c.559 A > G (p.Met187Val) and NM_000169.3(*GLA*):c.679 C > T (p.Arg227Ter), and a variant of unknown significance (VUS) reclassificated to a likely pathogenic variant NM_000169.3(*GLA*):c.122 C > G (p.Thr41Ser). Cascade screening identified three additional women with FD.

**Conclusion:**

These results emphasize the need for genetic screening for FD in female individuals with LVH. Further studies are needed to understand genotype-phenotype correlations in females with genetic variants in the *GLA* gene, particularly those with VUS and novel variants, as they pose challenges for diagnosis and treatment.

## Introduction

Fabry disease (FD) is an X-linked sphingolipidosis caused by pathogenic variants in the *GLA* gene. These genetic variants lead to a deficiency of enzyme α-Galactosidase A (α-Gal A) activity, causing accumulation of glycosphingolipids, particularly globotriaosylceramide (Gb3) and its derivative globotriaosylsohingosine (lyso-Gb3), in affected tissues [1]. Although any organ may be affected, cardiac involvement is the primary cause of reduced quality of life and mortality in patients with FD [2]. As a result of progressive glycosphingolipid deposits within all cardiac cell types — including cardiomyocytes, endothelial and smooth muscle cells, valvular fibroblasts, conduction tissue, and immune cells — significant cardiac dysfunction may develop over time [3, 4].

Early manifestations of FD are left ventricular hypertrophy (LVH) and diastolic dysfunction, which can be present even before symptoms appear [5]. This late onset of symptoms and heterogeneous clinical presentation contribute to frequent delays in the diagnosis of FD, hindering the timely initiation of disease-specific treatment [6]. Early detection of affected individuals is imperative, as particular therapy may limit disease progression and prevent irreversible organ damage [2, 7]. Furthermore, systematic screening of patients at high risk may help detect the disease promptly [8].

The estimated prevalence of FD in LVH patients is 0.9% [2]. Nonetheless, previous screening studies in such individuals have reported a prevalence ranging from 0 to 12%, identifying a variety of *GLA* gene variants [9]. These discrepancies may be related to the screening methods and eligibility criteria. This study aimed to describe the prevalence of FD in Mexican patients with LVH, a population that has not been previously explored.

## Methods

A single-center retrospective study was conducted between May 2022 and December 2023 in the cardiomyopathy clinic of the *Instituto Nacional de Cardiología Ignacio Chávez*, a tertiary hospital in Mexico City. Patients were recruited from a specialized cardiomyopathy clinic, where individuals with left ventricular hypertrophy (LVH) undergo comprehensive evaluation to exclude secondary causes of hypertrophy, including congenital heart disease, severe valvular disease, and hypertensive heart disease as the sole explanation for the phenotype. Therefore, patients included in this study were considered to have unexplained LVH after routine clinical assessment by experienced cardiomyopathy specialists.

All unrelated patients aged 18 or older with unexplained echocardiographic evidence of LVH, defined as LV wall thickness ≥ 12 mm and/or left ventricular mass index (LVMI) ≥ 95 g/m^2^ in women and ≥ 115 g/m^2^ in men [8], were screened for FD. The presence of hypertension or valvular heart disease was not considered an exclusion criterion when these conditions were not deemed sufficient to fully explain the degree or pattern of LVH after comprehensive clinical evaluation. Patients with a pathogenic variant in hypertrophic cardiomyopathy genes or any other inherited metabolic disorder that explains LVH were excluded from the study.

With prior informed consent obtained, α-Gal A activity was measured using the dry blood spot method in all male individuals. Patients with a positive dried blood spot testing (α-Gal A activity < 15.3µmol/L/h) and all female participants underwent *GLA* gene sequencing.

Further cascade genetic screening was performed on the relatives of patients diagnosed with FD. Plasma lyso-GB3 concentration was assessed in all patients. Demographic data, clinical characteristics, electrocardiographic parameters, and laboratory findings were collected in a standardized form for further analysis.

## Results

The study included 205 patients with LVH (48.5 ± 18.7 years). The mean age at diagnosis of LVH was 42.1 ± 19 years. Of these, 53% were male, 60% were classified as NYHA functional class I, and 21% were classified as NYHA functional class II. 43% of patients were asymptomatic at the time of screening. In symptomatic subjects, dyspnea was the most frequent symptom reported in 26% of cases. In symptomatic subjects, dyspnea was the most frequent symptom reported in 26% of cases. All patients had preserved left ventricular systolic function, with a mean LVEF of 63.5% (56–69%). The mean interventricular septum thickness in tele-diastole was 19.2 mm (15.6–23.4 mm).

Among study participants, Fabry disease was identified in 3 of 205 patients, corresponding to a prevalence of 1.46% (95%CI, 0.30%–4.22%), all of whom were female. Among female participants, Fabry disease was identified in 3 of 95 patients, corresponding to a prevalence of 3.16% (95%CI, 0.66%–8.95%). Sequencing of the *GLA* gene revealed three genetic variants: two pathogenic variants, NM_000169.3(*GLA*):c.559 A > G (p.Met187Val) and NM_000169.3(*GLA*):c.679 C > T (p.Arg227Ter), and a variant of unknown significance (VUS), NM_000169.3(*GLA*):c.122 C > G (p.Thr41Ser), that was reclassified as likely pathogenic. Cascade genetic screening performed on relatives of patients with FD identified segregation of the pathogenic *GLA* variants. The baseline and imaging characteristics of patients are described in Tables 1 and 2.

**Table 1:** General characteristics of study participants

	Value (*N* = 205)
**Demographics**	
Age, years	48.52 ± 18.75
Male gender, n (%)	110 (53.7%)
Age at diagnosis, years	42.09 ± 18.98
Family history of SCD, n (%)	49 (23.9%)
Hypertension, n (%)	66 (32.2%)
Diabetes Mellitus, n (%)	35 (17.1%)
Dyslipidemia, n (%)	56 (27.3%)
ICD	25 (12.2%)
Pacemaker	23 (11.2%)
**NYHA Class**, ***n*(%)**	
Class I	123 (60%)
Class II	44 (21.5%)
Class III	7 (3.4%)
Class IV	1 (0.5%)
**Symptoms at diagnosis**, ***n*(%)**	
Asymptomatic	89 (43.4%)
Syncope	27 (13.2%)
Dyspnea	53 (25.9%)
Palpitations	26 (12.7%)
Angina	36 (17.6%)
Lipothymia	16 (7.8%)
**α-Gal A enzyme activity**,** µmol/L/h**	15.21 ± 8.35

Abbreviations: SCD, sudden cardiac death; ICD, implantable cardioverter-defibrillator; NYHA, New York heart association; α-Gal A, alpha- galactosidase. Data are expressed as the number (%) or mean ± s.d

**Table 2:** Echocardiographic characteristics and parameters of study participants

	(*N* = 205)
**Diastolic Dysfunction, ** ***n*** ** (%)**	
None	15 (7.3%)
Grade I	53 (25.9%)
Grade II	73 (35.6%)
Grade III	11 (5.4%)
**Cardiac wall motion**, ***n*(%)**	
Normal	132 (64.4%)
Hypokinesis	21 (10.2%)
Akinesia/ Dyskinesis	8 (3.9%)
**Systolic Anterior Motion**, ***n*(%)**	59 (28.8%)
**Mitral Insufficiency**, ***n*(%)**	
None	47 (22.9%)
Mild	75 (36.6%)
Moderate	25 (12.2%)
Severe	13 (6.3%)
**Echocardiographic parameters**	
IVSd, mm	19.2 (15.6–23.4)
LVPWd, mm	12.8 (10.1–15.6)
LVIDd, mm	40.95 ± 7.94
LVIDs, mm	26.04 ± 8.71
RWT	0.72 (0.54–0.9)
LVEF, %	63.5 (56–69)
Peak E-wave, m/s	0.73 (0.61–0.89)
Peak A-wave, m/s	0.61 (0.44–0.78)
E/A Ratio	1.28 (0.85–1.69)
E/e´ Ratio	12 (8.9–16)
DT, m/s	224 (178-271.5)
LVOT	12.1 (6-57.2)
LAVI, mL/m ^2^	48 (37–62)
PASP, mm Hg	32 (26–39)
**Cardiac MRI parameters**	
LVIDd, mL	46.82 ± 7.3
LVIDs, mL	28 (24–33)
IVSd, mm	20 (17–25)
LVPWd, mm	10 (8–13)
LVEF, %	68 (60–73)
RVEF, %	54.86 ± 10.42

Abbreviations: IVSd, interventricular septum thickness; LVPWd, end-diastolic left ventricular posterior wall thickness; LVIDd, left ventricular internal diameter end diastole; LVIDs, left ventricular internal diameter end systole; RWT, relative wall thickness; LVEF, left ventricular ejection fraction; DT; deceleration time; LVOT, left ventricular outflow tract; LAVI, left atrial volume index; PASP, pulmonary artery systolic pressure; RVEF, right ventricular ejection fraction. Data are expressed as the number (%), M (Q1,Q3) or mean ± s.d

Patient A was referred to our institution due to asymmetric left ventricular hypertrophy. Her symptoms were palpitations, which were exacerbated by physical exertion. Echocardiography revealed asymmetric left ventricular hypertrophy without outflow tract obstruction. FD screening showed a missense heterozygous pathogenic variant in exon 4 of the *GLA* gene, NM_000169.3(*GLA*):c.559 A > G (p.Met187Val) and a plasma lyso-Gb3 concentration of 4.0 ng/ml (reference value: <1.8 ng/ml). She was referred to Nephrology and Ophthalmology for further evaluation, which ruled out nephrotic syndrome, tubular injury, and ophthalmologic pathology. Cascade screening was conducted, identifying the same pathogenic variant in her mother.

Patient B was referred to our hospital for asymmetric left ventricular hypertrophy without outflow tract obstruction, diagnosed six years ago, along with elevated free light chains, but a normal K/L ratio (kappa: 45.13 mg/L, lambda: 36.35 mg/L, K/L ratio: 1.24). Her father died of unexplained sudden cardiac death at 40 years old, and her daughter passed away from an unspecified congenital cardiovascular disease. During the physical examination, diffuse angiokeratomas were noted. Light chains and transthyretin amyloidosis (ATTR) were excluded. FD enzymatic test revealed a plasma lyso-Gb3 concentration of 3.9 ng/ml (reference value: <1.8 ng/ml). Genetic testing identified a well-documented nonsense heterozygous pathogenic genetic variant in exon 5 of the *GLA* gene, NM_000169.3(*GLA*):c.679 C > T (p.Arg227Ter). Family screening identified two asymptomatic female carriers, her daughter and sister, with the same pathogenic variant.

**Fig. 1:**
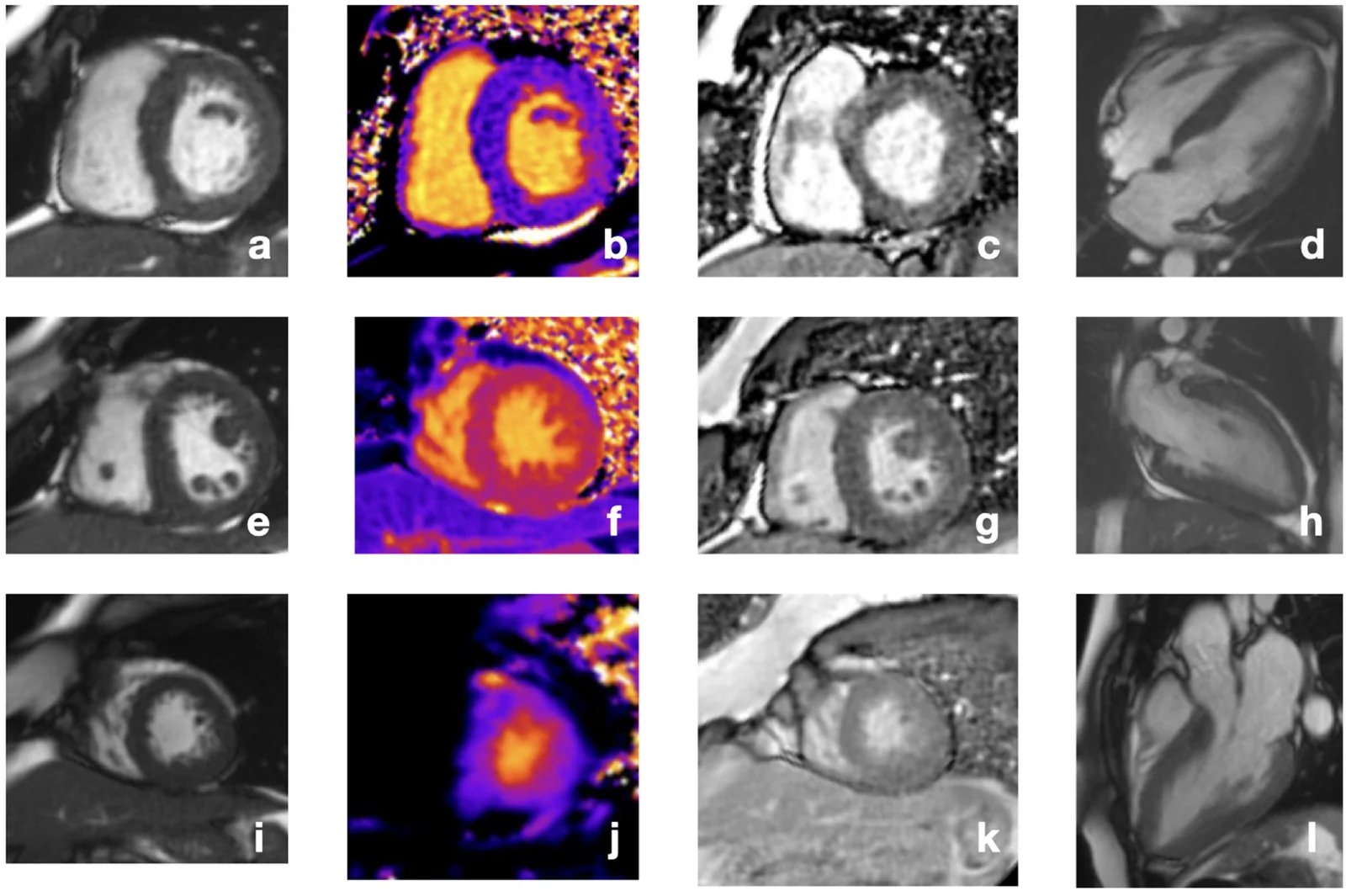
Magnetic resonance imaging short axis in basal (**a**-**c**), middle (**e**-**g**), and apical (**i**-**k**). In the first column, SSFP in diastole. The second column is with T1 mapping (933 ms), the third column is inversion-recovery. In (**d**) 4 chamber, (**h**) 2 chamber, and (**l**) 3 chamber

**Fig. 2:**
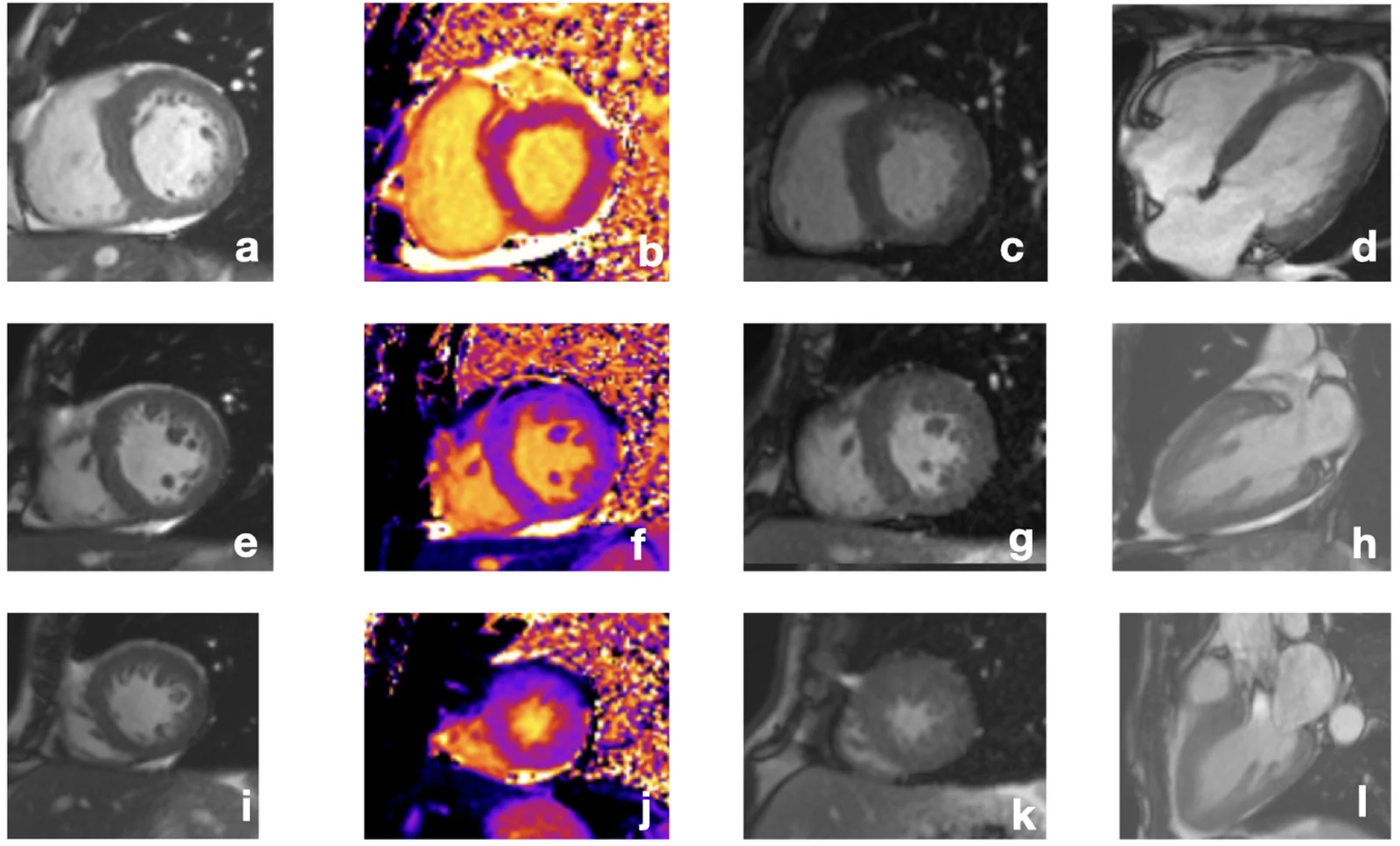
Magnetic resonance imaging short axis n basal (**a**-**c**), middle (**e**-**g**), and apical (**i**-**k**). In the first column, SSFP in diastole. The second column with T1 mapping (1040 ms), the third column is inversion-recovery. In (**d**) 4 chamber, (**h**) 2 chamber, and (**l**) 3 chamber in systole

Patient C was admitted to our facility for supraventricular tachycardia and hemodynamic instability. She was treated with 150 J electrical cardioversion and vasopressors. The patient had a history of a previous cerebrovascular event with a Modified Rankin Scale (MRS) grade of 0. One week before admission, she was diagnosed with a malignant cerebrovascular infarction MRS grade 4, followed by a right inferior segmental pulmonary thromboembolism. No thrombolysis therapy was received at the time. A neurological examination revealed left-sided paralysis and speech impairment. Due to the subject’s age and medical history, thrombophilia and antiphospholipid syndrome were suspected; however, screening was negative. Echocardiography revealed a restrictive left ventricular filling pattern and biatrial dilatation, with the posterior LV wall thickness measuring 13 mm. Cardiac Magnetic Resonance (CMR) imaging showed hyperintensities on the basal segment in T2-weighted sequences. Photon emission tomography (PET) and periumbilical biopsy were negative for sarcoidosis and amyloidosis. Given the multisystemic involvement, a suspicious diagnosis of FD was considered. The plasma lyso-Gb3 concentration was elevated at 4.5 ng/ml (reference value: <1.8 ng/ml). The genetic testing identified a missense heterozygous genetic variant in exon 1 of the *GLA* gene, NM_000169.3(*GLA*):c.122 C > G (p.Thr41Ser). This variant was initially reported as a VUS in the ClinVar database [9]. After applying the American College of Medical Genetics and Genomics and the Association for Molecular Pathology (ACMG/AMP) guidelines for variant interpretation [10], the variant was reclassified as likely pathogenic. The reclassification was based on weighted evidence from functional data, population data, protein effects, in silico predictions, and phenotype. The *GLA*:c.122 C > G variant fulfills ACMG/AMP criteria for likely pathogenic: PM1 (Pathogenic Moderate): non-truncating non-synonymous variant is located in a mutational or exonic hot spot. PM5 (Pathogenic Moderate): extremely low allele frequency (< 0.015%) in the gnomAD population database. PM5 (Pathogenic Moderate): a different amino acid change at the same codon is considered pathogenic. PP2 (Pathogenic Supporting): a missense variant in a gene with a low rate of benign missense mutations and for which a missense mutation is a common mechanism of a disease. PP3 (Pathogenic Supporting); computational prediction tools unanimously support a deleterious effect on the gene (REVEL score: 0.736). Phenotype: PP4 (Pathogenic Supporting); the patient’s phenotype is highly specific to the disease [9–15]. Cascade screening was negative for all relatives. Figures 1 and 2 show some of the findings observed in patients with Fabry disease.

## Discussion

The prevalence of FD among Mexican patients with LVH was 1.5%, which is highly consistent with previous studies conducted in the last decade (ranging from 0 to 12%) [16–24]. Although this is the first study to explore FD in Latin America, no significant differences were observed compared to other ethnic populations. This suggests that ethnicity does not play an important role in the disease’s pathogenicity, contrary to previous reports [25]. In this context, FD is a disease that affects all ethnic groups [26]. Similarly, a recent systematic review reported a pooled prevalence of 1.2% in patients with LVH after reevaluating and reclassifying *GLA* variants initially described as pathogenic/likely pathogenic (P/LP) as VUS/benign/likely benign (B/LB) and vice versa, according to the ACMG/AMP recommendations [10]. Haz clic o pulse aquí para escribir texto.However, the prevalence was 0.9% when using the ClinVar database criteria [9, 27]. These findings not only suggest a likely global prevalence regardless of ethnic origin, but they also highlight discrepancies in the classification of the clinical significance of *GLA* variants reported in the available literature.

Detecting new pathogenic variants through systematic screening of high-risk individuals is crucial for advancing our molecular understanding of the *GLA* gene and its clinical implications, especially in asymptomatic or mildly symptomatic patients [27, 28]. This approach could improve early diagnosis and enable more effective disease management. To date, over 1,000 variants of the *GLA* gene have been reported [29]. However, interpreting their pathogenicity requires extensive clinical and biochemical research, further complicating the already challenging disease [1, 26].

Our study found a heterozygous genetic variant, NM_000169.3(*GLA*):c.122 C > G (p.Thr41Ser), in a clinically affected 37-year-old woman with a high level of Lyso-Gb3. The genetic variant was initially reported as a VUS in the ClinVar database [9], but after applying the ACMG/AMP variant classification guidelines, the variant was reclassified as likely pathogenic. Although the *GLA*:c.122 C > G variant was recently identified in a newborn screening study for lysosomal storage disorders [30], our patient represents the first instance of severe multi-organ disease observed in an adult woman with this *GLA* gene variant. Genetic variants can be reclassified as new scientific evidence emerges regarding a given variant [27]. Accurate variant interpretation, which leads to a precise diagnosis, depends on up-to-date scientific evidence, standardized variant interpretation guidelines such as the ACMG/AMP recommendations [10], and collaboration among interdisciplinary teams, including medical geneticists, cardiologists, and others.

In order of the sex-specific diagnostic strategy used in this study, male participants were initially screened using α-galactosidase A activity, whereas all female participants underwent direct GLA gene sequencing. Although this approach reflects current clinical practice, it may have introduced detection bias. Consequently, the observation that all identified Fabry disease cases occurred in women should be interpreted with caution, as differences in diagnostic sensitivity between screening approaches may have influenced case detection.

Our findings highlight the importance of considering Fabry disease in female patients with unexplained LVH, particularly because enzyme-based screening strategies may be less reliable in heterozygous women. However, given the sex-specific diagnostic strategy used in this study and the limited number of identified cases, further studies are needed to determine whether the observed predominance of female cases reflects the true distribution of disease in this population.

Furthermore, the range of clinical symptoms depends on the specific *GLA* gene variant and the inactivation pattern of the X chromosome across different tissues. As a result, female patients may experience more severe symptoms or remain entirely asymptomatic [31, 32]. However, a notable increase in the occurrence of LVH and a decline in kidney function have been observed in asymptomatic female individuals, typically around a mean age of 50 years [33].Haz clic o pulse aquí para escribir texto.Thus, asymptomatic female patients may be at risk of developing severe FD cardiomyopathy or nephropathy by the time the disease is detected, mainly due to delayed diagnosis. This was particularly evident in all index cases in our study, as several years had elapsed between symptom onset and diagnosis. Likewise, all patients were initially misdiagnosed, resulting in a significant delay in starting appropriate treatment.

This study has several limitations. First, it was conducted at a single tertiary referral center, which may limit the generalizability of the findings to other populations and healthcare settings. Second, despite screening 205 patients with LVH, only three index cases of Fabry disease were identified, limiting the ability to draw robust conclusions regarding clinical characteristics and sex-specific prevalence. Third, because participants were recruited from a specialized cardiomyopathy clinic, referral and selection biases cannot be excluded. Finally, the use of different diagnostic strategies according to sex—enzyme activity screening in males and universal GLA sequencing in females—may have introduced detection bias and influenced the observed distribution of Fabry disease cases within the cohort.

## Conclusion

These results support the consideration of Fabry disease screening in patients with unexplained LVH. Identification of pathogenic GLA variants may facilitate cascade family screening and timely initiation of disease-specific therapies. However, larger multicenter studies are needed to better define the prevalence of Fabry disease and characterize sex-specific diagnostic performance in this population.

## Data Availability

All data generated or analyzed during this study are included in this published article. Any extra information is available from the corresponding author on reasonable request.

## References

[1] Germain DP, Altarescu G, Barriales-Villa R, Mignani R, Pawlaczyk K, Pieruzzi F, et al. An expert consensus on practical clinical recommendations and guidance for patients with classic fabry disease. Mol Genet Metab [Internet]. 2022 Sep 1 [cited 2025 Jun 19];137(1–2):49–61. Available from: https://pubmed.ncbi.nlm.nih.gov/35926321/. 10.1016/j.ymgme.2022.07.010 35926321

[2] Pieroni M, Moon JC, Arbustini E, Barriales-Villa R, Camporeale A, Vujkovac AC, et al. Cardiac involvement in fabry disease: JACC review topic of the week. J Am Coll Cardiol [Internet]. 2021 Feb 23 [cited 2025 Jun 24];77(7):922–36. Available from: https://pubmed.ncbi.nlm.nih.gov/33602475/. 10.1016/j.jacc.2020.12.024 33602475

[3] Azevedo O, Cordeiro F, Gago MF, Miltenberger-miltenyi G, Ferreira C, Sousa N, et al. Fabry disease and the heart: a comprehensive review. Int J Mol Sci [Internet]. 2021 May 1 [cited 2025 Jun 24];22(9). Available from: https://pubmed.ncbi.nlm.nih.gov/33922740/. 10.3390/ijms22094434 PMC8123068 33922740

[4] Weissman D, Dudek J, Sequeira V, Maack C. Fabry disease: cardiac implications and molecular mechanisms. Curr Heart Fail Rep [Internet]. 2024 Apr 1 [cited 2025 Jun 24];21(2):81–100. Available from: https://pubmed.ncbi.nlm.nih.gov/38289538/. 10.1007/s11897-024-00645-1 PMC10923975 38289538

[5] Aquaro GD, De Gori C, Faggioni L, Parisella ML, Aringhieri G, Cioni D, et al. Cardiac magnetic resonance in fabry disease: morphological, functional, and tissue features. Diagnostics [Internet]. 2022 Nov 1 [cited 2025 Jun 24];12(11). Available from: https://pubmed.ncbi.nlm.nih.gov/36359495/. 10.3390/diagnostics12112652 PMC9689455 36359495

[6] Esposito P, Caputo C, Repetto M, Somaschini A, Pietro B, Colomba P, et al. Diagnosing fabry nephropathy: the challenge of multiple kidney disease. BMC Nephrol [Internet]. 2023 Dec 1 [cited 2025 Jun 24];24(1). Available from: https://pubmed.ncbi.nlm.nih.gov/37990184/. 10.1186/s12882-023-03388-8 PMC10664682 37990184

[7] Umer M, Kalra DK. Treatment of fabry disease: established and emerging therapies. Pharmaceuticals. 2023 Feb 20 [cited 2025 Jun 24];16(2):320. Available from: https://www.mdpi.com/1424-8247/16/2/320/htm. 10.3390/ph16020320 PMC9967779 37259462

[8] Regenbogen C, Braunisch MC, Schmaderer C, Heemann U. Fabry disease: What the cardiologist should consider in non-cardiac screening, diagnosis, and management—narrative review. Cardiovasc Diagn Ther [Internet]. 2021 Apr 1 [cited 2025 Jun 24];11(2):661–71. Available from: https://pubmed.ncbi.nlm.nih.gov/33968643/. 10.21037/cdt-20-845 PMC8102250 33968643

[9] Landrum MJ, Lee JM, Riley GR, Jang W, Rubinstein WS, Church DM, Maglott DR. ClinVar: public archive of relationships among sequence variation and human phenotype. Nucleic Acids Res. 2014;42(Database issue):D980–5. 10.1093/nar/gkt1113. PMC3965032 24234437

[10] Richards S, Aziz N, Bale S, Bick D, Das S, Gastier-Foster J, Grody WW, Hegde M, Lyon E, Spector E, Voelkerding K, Rehm HL, ACMG Laboratory Quality Assurance Committee. Standards and guidelines for the interpretation of sequence variants: a joint consensus recommendation of the American College of Medical Genetics and Genomics and the Association for Molecular Pathology. Genet Med. 2015;17(5):405–24. 10.1038/gim.2015.30. PMC4544753 25741868

[11] Bergquist T, Stenton SL, Nadeau EAW, Byrne AB, Greenblatt MS, Harrison SM, Tavtigian SV, O’Donnell-Luria A, Biesecker LG, Radivojac P, Brenner SE, Pejaver V. Calibration of additional computational tools expands ClinGen recommendation options for variant classification with PP3/BP4 criteria. Genet Med. 2025;27(6):101402. 10.1016/j.gim.2025.101402. PMC12208618 40084623

[12] Shabbeer J, Yasuda M, Benson SD, Desnick RJ. Fabry disease: identification of 50 novel alpha-galactosidase A mutations causing the classic phenotype and three-dimensional structural analysis of 29 missense mutations. Hum Genomics. 2006;2(5):297–309. 10.1186/1479-7364-2-5-297. PMC3500179 16595074

[13] X-100662770-G-C. gnomAD V2.1.1, gnomAD. 2026. https://gnomad.broadinstitute.org/variant/X-100662770-G-C?dataset=gnomad_r2_1.

[14] ClinGen Allele Registry, ClinGen Clinical Genome Resource. 2026. https://reg.clinicalgenome.org/redmine/projects/registry/genboree_registry/by_caid?caid=CA029760.

[15] rs782362194 RefSNP Report - dbSNP - NCBI. 2026. https://www.ncbi.nlm.nih.gov/snp/rs782362194.

[16] Citro R, Prota C, Ferraioli D, Iuliano G, Bellino M, Radano I, et al. Importance of echocardiography and clinical red flags in guiding genetic screening for fabry disease. Front Cardiovasc Med [Internet]. 2022 Apr 25 [cited 2025 Jun 24];9. Available from: https://pubmed.ncbi.nlm.nih.gov/35548424/. 10.3389/fcvm.2022.838200 PMC9081601 35548424

[17] Terryn W, Deschoenmakere G, De Keyser J, Meersseman W, Van Biesen W, Wuyts B, et al. Prevalence of fabry disease in a predominantly hypertensive population with left ventricular hypertrophy. Int J Cardiol [Internet]. 2013 [cited 2025 Jun 24];167(6):2555–60. Available from: https://pubmed.ncbi.nlm.nih.gov/22805550/. 10.1016/j.ijcard.2012.06.069 22805550

[18] Kubo T, Ochi Y, Baba Y, Hirota T, Tanioka K, Yamasaki N, et al. Prevalence and clinical features of fabry disease in Japanese male patients with diagnosis of hypertrophic cardiomyopathy. J Cardiol [Internet]. 2017 Jan 1 [cited 2025 Jun 24];69(1):302–7. Available from: https://pubmed.ncbi.nlm.nih.gov/27554049/. 10.1016/j.jjcc.2016.05.014 27554049

[19] Cecchi F, Iascone M, Maurizi N, Pezzoli L, Binaco I, Biagini E, et al. Intraoperative diagnosis of Anderson-Fabry disease in patients with obstructive hypertrophic cardiomyopathy undergoing surgical myectomy. JAMA Cardiol [Internet]. 2017 Oct 1 [cited 2025 Jun 24];2(10):1147–51. Available from: https://pubmed.ncbi.nlm.nih.gov/28793143/. 10.1001/jamacardio.2017.2353 PMC5815007 28793143

[20] Barman HA, İkitimur B, Avcı BK, Durmaz E, Atıcı A, Aslan S, et al. The prevalence of fabry disease among Turkish patients with non-obstructive hypertrophic cardiomyopathy: insights from a screening study. Balkan Med J [Internet]. 2019 Nov 1 [cited 2025 Jun 24];36(6):354–8. Available from: https://pubmed.ncbi.nlm.nih.gov/31446751/. 10.4274/balkanmedj.galenos.2019.2019.5.125 PMC6835159 31446751

[21] Sadasivan C, Chow JTY, Sheng B, Chan DKH, Fan Y, Choi PCL, et al. Screening for Fabry disease in patients with unexplained left ventricular hypertrophy. PLoS ONE [Internet]. 2020 Sep 1 [cited 2025 Jun 24];15(9). Available from: https://pubmed.ncbi.nlm.nih.gov/32987398/. 10.1371/journal.pone.0239675 PMC7521938 32987398

[22] Xiao Y, Sun Y, Tian T, Wang TJ, Zhao RX, Zhang Y, et al. Prevalence and clinical characteristics of Fabry disease in Chinese patients with hypertrophic cardiomyopathy. Am J Med Sci. 2021 Sep 1 [cited 2025 Jun 24];362(3):260–7. Available from: https://pubmed.ncbi.nlm.nih.gov/34266644/. 10.1016/j.amjms.2021.01.009 34266644

[23] Savostyanov K, Pushkov A, Zhanin I, Mazanova N, Trufanov S, Pakhomov A, et al. The prevalence of Fabry disease among 1009 unrelated patients with hypertrophic cardiomyopathy: a Russian nationwide screening program using NGS technology. Orphanet J Rare Dis [Internet]. 2022 Dec 1 [cited 2025 Jun 24];17(1):1–8. Available from: https://ojrd.biomedcentral.com/articles/10.1186/s13023-022-02319-4. 10.1186/s13023-022-02319-4 PMC9109305 35578305

[24] Zemánek D, Januška J, Honěk T, Čurila K, Kubánek M, Šindelářová Š, et al. Nationwide screening of Fabry disease in patients with hypertrophic cardiomyopathy in Czech Republic. ESC Heart Fail [Internet]. 2022 Dec 1 [cited 2025 Jun 24];9(6):4160–6. Available from: https://pubmed.ncbi.nlm.nih.gov/36087038/. 10.1002/ehf2.14135 PMC9773751 36087038

[25] Hsu TR, Hung SC, Chang FP, Yu WC, Sung SH, Hsu CL, et al. Later onset Fabry disease, cardiac damage progress in silence: experience with a highly prevalent mutation. J Am Coll Cardiol [Internet]. 2016 Dec 13 [cited 2025 Jun 24];68(23):2554–63. Available from: https://pubmed.ncbi.nlm.nih.gov/27931613/. 10.1016/j.jacc.2016.09.943 27931613

[26] Gragnaniello V, Burlina AP, Commone A, Gueraldi D, Puma A, Porcù E, et al. Newborn screening for Fabry disease: current status of knowledge. Int J Neonatal Screen [Internet]. 2023 Jun 1 [cited 2025 Jun 24];9(2). Available from: https://pubmed.ncbi.nlm.nih.gov/37367212/. 10.3390/ijns9020031 PMC10299185 37367212

[27] Monda E, Diana G, Graziani F, Rubino M, Bakalakos A, Linhart A, et al. Impact of GLA variant classification on the estimated prevalence of Fabry disease: a systematic review and meta-analysis of screening studies. Circ Genom Precis Med [Internet]. 2023 Dec 1 [cited 2025 Jun 24];16(6):E004252. Available from: 10.1161/CIRCGEN.123.004252?download=true. 38047356

[28] Sawada T, Kido J, Sugawara K, Nakamura K. High-risk screening for Fabry disease: a nationwide study in Japan and literature review. Diagnostics. 2021 Sep 27 [cited 2025 Jun 24];11(10):1779. Available from: https://www.mdpi.com/2075-4418/11/10/1779/htm. 10.3390/diagnostics11101779 PMC8534369 34679477

[29] Sezer O, Ceylaner S. Genetic management algorithm in high-risk Fabry disease cases; especially in female indexes with mutations. Endocr Metab Immune Disord Drug Targets [Internet]. 2020 Jul 9 [cited 2025 Jun 24];21(2):324–37. Available from: https://pubmed.ncbi.nlm.nih.gov/32640971/. 10.2174/1871530320666200708135826 32640971

[30] Sanders KA, Gavrilov DK, Oglesbee D, Raymond KM, Tortorelli S, Hopwood JJ, et al. A comparative effectiveness study of newborn screening methods for four lysosomal storage disorders. Int J Neonatal Screen [Internet]. 2020 Jun 1 [cited 2025 Jun 24];6(2). Available from: https://pubmed.ncbi.nlm.nih.gov/32802993/. 10.3390/ijns6020044 PMC7423013 32802993

[31] Izhar R, Borriello M, La Russa A, Di Paola R, De A, Capasso G, et al. Fabry disease in women: genetic basis, available biomarkers, and clinical manifestations. Genes (Basel) [Internet]. 2024 Jan 1 [cited 2025 Jun 24];15(1). Available from: https://pubmed.ncbi.nlm.nih.gov/38254927/. 10.3390/genes15010037 PMC10815601 38254927

[32] Hopkin RJ, Cabrera GH, Jefferies JL, Yang M, Ponce E, Brand E, et al. Clinical outcomes among young patients with Fabry disease who initiated agalsidase beta treatment before 30 years of age: an analysis from the Fabry registry. Mol Genet Metab [Internet]. 2023 Feb 1 [cited 2025 Jun 24];138(2). Available from: https://pubmed.ncbi.nlm.nih.gov/36709533/. 10.1016/j.ymgme.2022.106967 36709533

[33] Wanner C, Feldt-Rasmussen U, Jovanovic A, Linhart A, Yang M, Ponce E, et al. Cardiomyopathy and kidney function in agalsidase beta-treated female Fabry patients: a pre-treatment vs. post-treatment analysis. ESC Heart Fail [Internet]. 2020 Jun 1 [cited 2025 Jun 24];7(3):825–34. Available from: https://pubmed.ncbi.nlm.nih.gov/32100468/. 10.1002/ehf2.12647 PMC7261571 32100468

